# Geocoding of worldwide patent data

**DOI:** 10.1038/s41597-019-0264-6

**Published:** 2019-11-06

**Authors:** Gaétan de Rassenfosse, Jan Kozak, Florian Seliger

**Affiliations:** 1 Chair of Innovation and Intellectual Property Policy, College Management of Technology, Ecole polytechnique fédérale de Lausanne, Lausanne, Switzerland; 2 KOF Swiss Economic Institute, Department of Management, Technology, and Economics, ETH Zurich, Zurich, Switzerland

**Keywords:** Economics, Intellectual-property rights, Databases

## Abstract

The dataset provides geographic coordinates for inventor and applicant locations in 18.8 million patent documents spanning over more than 30 years. The geocoded data are further allocated to the corresponding countries, regions and cities. When the address information was missing in the original patent document, we imputed it by using information from subsequent filings in the patent family. The resulting database can be used to study patenting activity at a fine-grained geographic level without creating bias towards the traditional, established patent offices.

## Abstract (summary)

**Table:** 

Measurement(s)	patent • geographic location
Technology Type(s)	digital curation • geocoding
Factor Type(s)	geographic location • source • year

Machine-accessible metadata file describing the reported data: 10.6084/m9.figshare.9970454

## Background & Summary

Patents are jurisdictional rights, and applicants willing to protect an invention internationally must file individual patent applications in all countries where they seek protection. The patent document that first describes the invention is usually called the ‘priority filing’ or ‘first filing’ (or priority patent application) and the patent documents subsequently filed in other jurisdictions are called ‘second filings.’ In 2010, there were 2.5 million patent applications filed worldwide. About 1 million of these were first filings, i.e. new inventions submitted for patent protection—the rest was second filings.

The goal of this project has been to produce a dataset of first filings filed across the globe and to allocate them by inventor and applicant location. The patent data relate exclusively to invention patents, and exclude, e.g., plant patents and designs (sometimes called utility models or petty patents). For example, the database allows identifying all patented inventions by inventors located in Switzerland (or in a specific region in Switzerland), regardless of the patent office at which the applications are filed. In many academic studies and policy reports, patenting activity is measured at a single patent office such as the European Patent Office (EPO) or the United States Patent and Trademark Office (USPTO)^1,2^. But these offices only attract a selected set of patented inventions. For example, our data suggest that less than 30 percent of all first filings by Swiss inventors in 2010 were filed at the EPO.

Obtaining precise geographic information is important for several reasons. First, since knowledge spillovers are concentrated locally and decay fast with geographical distance^3,4^, a high level of granularity is desirable for such studies. Second, innovative activity is usually distributed very unequally within countries and a small number of cities and regions account for most of the patent applications^5^. In addition, the worldwide coverage of the data enables institutional research on innovation activities in emerging or developing economies^6^, which are overlooked in the literature. Third, policymakers are increasingly interested in location decisions of firms and high-skilled labor^7^. For this purpose, it is important to know where the major innovation hubs are located. Geographic coordinates allow for a variety of geographic distance calculations, spatial clustering and visualizations. Accurate geolocation data also serve other purposes such as tracking inventor mobility^8^ or improving disambiguation algorithms^9^.

The present work draws on the Worldwide Count of Priority Patent Applications (WCPPA) proposed in de Rassenfosse *et al*.^10^, but extends it both in scope and in depth. First, whereas the original work exploited information from one patent database (PATSTAT), we have combined information from nine national, regional and international patent databases. Oftentimes, address information is missing from the PATSTAT database and the consideration of several databases has improved data coverage substantially. Second, we provide a more fine-grained localization measure. Whereas the original work allocated patents to inventor and applicant countries, the present work allocates patents to precise geographic coordinates using the full address information. It also assigns each address to corresponding countries, regions and cities. The resulting dataset enables researchers to identify, say, how many inventions (that is, first filings) related to chemistry were produced in the area of Kanpur, India in year 2010. Overall, we have collected and geocoded 7 million inventor and applicant addresses from 18.8 million first filings invented in 46 countries and filed between 1980 and 2014. The dataset has a high coverage: it covers 81 percent of all first filings applied for across the globe over the considered time period.

Researchers focusing on regions traditionally use the OECD REGPAT database, which offers a regional breakdown of patent applications filed at the EPO and at the World Intellectual Property Organization (WIPO)^11^. REGPAT also provides information on postal codes and city names from the addresses listed in the patent documents at these two offices. This database has been widely used in academic studies^12–15^ and policy reports^16,17^, yet it represents a selected set of patents that reflects a fraction of overall patenting activity. The present dataset considerably expands REGPAT. It enables counting the number of first filings for 54,583 cities across the globe and for administrative divisions at different levels of precision (e.g., regions, departments, arrondissements, and communes in France or states (*Länder*), governmental districts, districts (*Kreise*), and municipalities in Germany). The Supplementary File 1 provides a table that offers a more systematic comparison between the present work and earlier works.

**Fig. 1:**
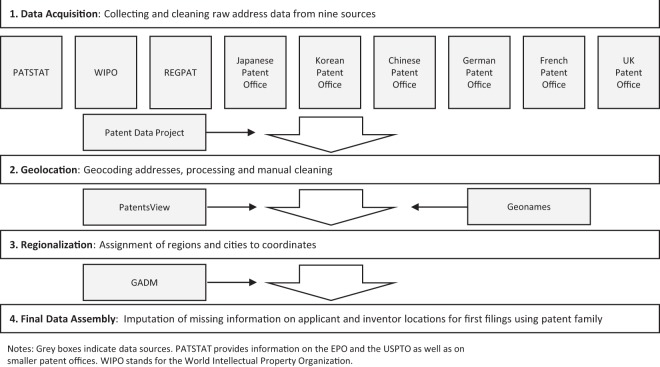
Schematic Overview of the Data Generation Process.

Figure 1 provides an overview of the data generation process, which consists of four main stages. Stage 1 relates to the acquisition and cleaning of address data from several data sources. The objective is to prepare a list of all addresses available in the relevant patent documents to be used for geolocation. These patent documents do not all correspond to first filings. They also include second filings. Stage 2 concerns the geolocation. We feed all addresses to a commercial geolocation service and perform extensive cleaning of the returned results. We replenish ambiguous, missing and wrong information with external data (Geonames and PatentsView). Stage 3 involves the regionalization. We allocate all addresses to a country, a city and administrative area(s). Stage 4 deals with final data assembly. We assign all patent documents into the families to which they belong, and use family information to find the address for the first filings. Extensive data quality checks suggest that the information provided is highly accurate overall. Data quality for China could be improved with better raw data becoming available from official sources.

The resulting dataset includes all first filings and the associated geographic information. The data are distributed as a set of txt files that have been designed for easy interoperability with two major patent databases. The dataset natively connects to the PATSTAT database, and the data release also includes a bridge table to connect to USPTO’s database PatentsView and other national patent databases.

Note that the datasets do not contain information on individual inventors and applicants. They contain geolocation data for inventors and applicants but do not allow identifying actual individual inventors and applicants. Sometimes, address data are missing in the original patent documents and we recover them from subsequent filings—this trick prevents us from linking inventors and applicants listed in the original patent documents to their addresses in a consistent manner. Furthermore, disambiguating inventors and applicants is a particularly challenging task and forms a research project on its own^8^.

The remainder of this document describes the four steps of the data generation process in detail (Methods section), presents the organization of the final dataset and discusses coverage (Data Records), explores data quality (Technical Validation) and provides usage information (Usage Notes).

## Methods

### Stage 1: data acquisition

The data collection involved building a large database of patent filings along with their corresponding applicant and inventor addresses from the major patent offices. We focus on 46 inventor and applicant countries that represent all OECD, EU28 and BRICS countries and account for more than 99 percent of all first filings in PATSTAT that were filed between 1980 and 2014 (see Table 1). Extending the database to additional, smaller countries would have increased the cost of data processing considerably for a marginal improvement in coverage.

**Table 1:** Included inventor and applicant countries, and corresponding country codes.

Country code	Country	Country code	Country
AU	Australia	LV	Latvia
AT	Austria	LI	Liechtenstein
BE	Belgium	LT	Lithuania
BR	Brazil	LU	Luxembourg
BG	Bulgaria	MT	Malta
CA	Canada	MX	Mexico
CL	Chile	NL	Netherlands
CN	China	NZ	New Zealand
HR	Croatia	NO	Norway
CZ	Czech Republic	PL	Poland
DK	Denmark	PT	Portugal
EE	Estonia	RO	Romania
FI	Finland	RU	Russian Federation
FR	France	SK	Slovak Republic
DE	Germany	SI	Slovenia
GR	Greece	ZA	South Africa
HU	Hungary	KR	South Korea
IS	Iceland	ES	Spain
IN	India	SE	Sweden
IE	Ireland	CH	Switzerland
IL	Israel	TR	Turkey
IT	Italy	GB	United Kingdom
JP	Japan	US	United States

We started with address data that are available in the PATSTAT database (autumn 2016 version). PATSTAT is maintained by the EPO and contains bibliographical and legal status data from patent offices of leading industrialized and developing countries^18^. PATSTAT provides addresses from patent applications filed at the EPO and city names from patent applications filed at the USPTO. It also contains address information for other, smaller patent offices but with varying degrees of coverage.

In a second step, we sought to fill gaps in addresses by adding information directly obtained from other patent offices. We added address information for international patent applications filed under the Patent Cooperation Treaty (so-called PCT filings) from a dataset obtained directly from WIPO. This dataset contains full address information for patents originating from European countries. Addresses for PCT applications originating from outside Europe were obtained from the OECD REGPAT database. However, only postal codes and city names are available in REGPAT. We then gathered address data from six national patent offices: China, France, Germany, Japan, South Korea and the United Kingdom.

Note that the combination of these databases allows us to obtain address information for first filings from a large number of patent offices even if the information from a particular office is not included directly. When address information for, say, a patent application filed at the Swiss patent office is not available in PATSTAT, it is possible to recover it from, say, a second filing that was submitted to the German patent office. (If the Swiss applicant also sought protection in Germany, of course.) This method of imputation of missing data was first proposed in de Rassenfosse *et al*. (2013) and has been shown to perform well.

All datasets either contain a patent publication number or a patent application number. These numbers are issued by the patent offices and can be found also in PATSTAT. Therefore, we were able to connect all data to PATSTAT tables and to assign a PATSTAT identifier (appln_id). Regarding the Japanese and Korean offices, minor alterations to the application numbers both in PATSTAT and in the patent office data were necessary before joining them with PATSTAT tables.

Below, we present the data sources used to build the present dataset and explain how we process them.

### Data sources

#### PATSTAT

The address fields are available in PATSTAT tables TLS906_PERSON and TLS226_PERSON_ORIG. The former contains the whole address field (person_address) and the latter contains only substrings of this field (labelled address_1 to address_5) where whitespaces or commas have been used as separators. For some countries, only the last two or three substrings contain address information as the preceding substrings often contain information on companies and research institutes that we did not use for localization. Table 2 provides selected examples. We manually checked a random sample of addresses for each country in order to see how many substrings were needed to identify the location accurately. We used two to three substrings for the following countries: Belgium, Finland, France, Hungary, India, Italy, Latvia, Netherlands, Norway, Poland, Slovak Republic, Slovenia, Sweden, Switzerland and the United States. We used the last substring in the case of Malta. Finally, we used all information in the person_address field for the remaining countries.

**Table 2:** Selected examples of address fields in PATSTAT.

person_address	address_1	address_2	address_3	address_4	address_5
Janssen Pharmaceutica N.V., Turnhoutseweg 30, B-2340 Beerse	Janssen Pharmaceutica N.V.	Turnhoutseweg 30	B-2340 Beerse		
Université de Geneve (UNIGE), Faculty of Medicine, Dept. of Pathology and Immunology, 1 Rue Michel Servet,CH-1211 Geneva	Université de Geneve (UNIGE)	Faculty of Medicine	Dept. of Pathology and Immunology	1 Rue Michel Servet	CH-1211 Geneva
John F. Welch Technology Centre Pvt. Ltd., Plot 22, EPIP, Phase II, Hoodi Village, Whitefield Road, 560066 Bangalore, Karnataka	John F. Welch Technology Centre Pvt. Ltd,	Plot 22, EPIP, Phase II, Hoodi Village	Whitefield Road	560066 Bangalore, Karnataka	

#### World intellectual property organization (WIPO)

Address data for PCT filings with inventor or applicant locations in Europe come from a dataset we obtained from WIPO. We used the address field that was available without modification.

#### REGPAT

The OECD REGPAT database contains postal codes and city names for PCT filings. We used information from this database for all inventor and applicant locations that could not be recovered from the WIPO dataset.

#### German patent and trademark office (DPMA)

Obtaining data from the *Deutsches Patent- und Markenamt* (DPMA) required signing a formal data contract between DPMA and the university. The data are free of charge for public research institutes and are available on a DVD-ROM in multiple zipped files in XML format. The files cover patent applications filed between 1877 and 2016, but inventor information is only available starting from 1988.

The fields on inventors and applicants contain name and address information in a single string. Address data include cities and postal codes; no detailed address information is available. The data are more complete for German inventors/applicants than for foreigners. Table 3 provides two typical examples:

**Table 3:** Selected examples of address fields in the DPMA data.

Type	Example of typical field
Inventor	Mospak, Christian, Dipl.-Ing. (FH), 71069 Sindelfingen
Applicant	SICAN Gesellschaft für Silizium-Anwendungen und CAD/CAT Niedersachsen mbH, 30419 Hannover

After parsing the XML files, we extracted the address information from the inventor and applicant fields. For that purpose, we split the string using the comma as separator. We constructed the address field as follows:

1. The last substring identifies the city in almost all cases.
2. For some countries such as the United Kingdom or Canada, the last substring sometimes identifies a region; in such cases, the preceding substring (second from the end) identifies the city.
3. A postal code was added to the address field if the substring preceding the city is numeric.

#### French national industrial property institute (INPI)

Obtaining data from the *Institut National de la Propriété Industrielle* (INPI) required signing a license agreement to reuse the data. The data were delivered free of charge through FTP access in zipped XML files covering the years 1985–2016. Because the data coverage did not seem to be complete, we complemented the data with data obtained previously covering the years 1997 to 2004 (de Rassenfosse *et al*. 2013).

The files contain separate fields for street, city and postal code for both inventors and applicants. We combined these fields to create individual address fields containing street, city and postal code for applicants and inventors.

#### United Kingdom intellectual property office (UK IPO)

Data from the UK IPO was provided as a zipped CSV file by email, free of charge. The file includes information for all published patent applications since 1978. No pre-processing of addresses was needed: The file came with columns for inventor and applicant addresses. The inventor can demand to not release her address. In such rare cases it was suppressed from the dataset we received.

#### Korean intellectual property office (KIPO)

The KIPO disseminates information via its internet-based service ‘KIPRIS,’ which stands for Korean Intellectual Property Rights Information Service. The platform enables document searches free of charge but also makes the data available for download for a fee. Different download options are available, including an application programming interface (API), which we used. The KIPRIS team kindly agreed for a one-time exceptional waiver of the fee to support our research.

We followed a two-step procedure to obtain the data. First, we obtained patent application numbers for a range of dates. Second, we fed the patent application numbers to the API to get address data in XML format. The data cover the years from 1980 to mid-2017.

We deleted all non-Asian countries from the data because almost all non-Asian applicants and inventors at KIPO also file their patents at the USPTO or at the EPO, for which we have good data in PATSTAT. By preferring data from Western patent offices for Western countries we avoided problems arising from translating addresses back and forth between Latin and Hangul characters. In order to delete non-Asian countries, we had to identify all country words in the addresses in Korean using Google Translate. Finally, we also deleted data from Japanese inventors and applicants, for which we have good data (see below).

#### Japanese patent office (JPO)

Addresses from the JPO could be downloaded as CSV files from a bulk data homepage (http://www.iip.or.jp/e/patentdb/index.html) for the application years 1963 until 2013^19^. Separate files are available for applicants and inventors. The application numbers from PATSTAT required some manipulation in order to match with the number in the JPO data. More concretely, each application number must have the application year at the beginning and 10 digits in total. Some digits in between needed to be filled with zeroes to correspond with the JPO number.

We did some heavy processing on the Japanese data. We checked a large number of addresses with Google Translate and it turns out that many addresses outside Japan are misspelled. The JPO applicant table contains a country code that does not always correspond to the information in the address field. Therefore, we identified the country ourselves with the information given in the address field. We noticed several spelling variants for countries in the address fields. We identified most of the spelling variants by inserting a large number of addresses into Google Translate in order to identify the part of the address that identifies the country. We repeated this process and built a comprehensive dictionary of spelling variants associated with countries. At the end, we could identify the country in 95.5% of the inventor addresses from JPO. Again, we deleted all non-Asian addresses and also Korean addresses.

#### China national intellectual property administration (CNIPA)

Chinese patent data are relatively difficult to obtain—in essence, CNIPA requires a contractual agreement with an entity before sharing patent data and the entity must be based in China. There are some commercial services available, but the cost is excessive and data quality is unknown. Instead, we were able to obtain CSV files with address data from the first applicant at CNIPA from Chinese colleagues. Data on inventor addresses are not available at the moment^20^. The data cover the years 1984 to 2013. As addresses were still partly missing, we complemented the data with addresses for listed firms from the Chinese Patent Project that are available for the years 1998 to 2009^21^.

The address fields contain country codes, which we used to exclude all non-Asian countries plus Japan and Korea. Most of the addresses contain a 6-digit postal code at the beginning.

### Data preparation

Although all patent offices require inventor and applicant addresses at the time a patent is filed, the data coverage and quality released to the public differ widely across (and within) patent offices. The address data in patent documents have many inconsistencies, spelling mistakes and coverage problems. We sought to harmonize the data with two objectives in mind: improve the usability of the data, and thereby minimize errors during the geolocation process in Stage 2; and reduce the number of addresses to geolocate (and thus lower processing cost). As the data structure depends on the patent office’s practices (in the case of Asian offices, the language is a complicating factor), we had to consider many special cases. The cleaning was implemented in PostgreSQL. The set of heuristic rules and more details on the cleaning procedures that we applied to all addresses can be found in the Supplementary File 2. After applying all procedures, we ended up with a database of 7 million unique addresses. The data were stored in two tables:

1. Addresses_patstat with about 4.5 million ‘unique’ addresses from PATSTAT and REGPAT (and WIPO) containing cleaned addresses from the USPTO, the EPO, the WIPO, and from national offices (if available in PATSTAT).
2. Addresses_further_data with about 2.5 million ‘unique’ addresses from further European and Asian offices.

Although we did our best to remove inconsistencies in the cleaning procedures, we have to accept that there are still addresses that are in fact duplicates, e.g., if one address contains a spelling mistake. However, many of these cases will be handled during the geocoding.

The technical validation section discusses the validation of the address cleaning.

### Stage 2: geolocation

In order to geocode all 7 million addresses, we used commercial online web services (see, e.g., https://geopy.readthedocs.io/en/latest/#module-geopy.geocoders). Such services can geolocate addresses all over the world in many languages, which is very convenient for us because the Asian data are in Korean, Japanese and Chinese languages. They can also handle typical problems such as spelling mistakes and inconsistent formatting to a certain degree. We have developed a program that queries the addresses from a database, queries each address in their APIs, parses it and saves the result to the database.

Geolocalization services use machine learning for their searching and matching algorithms in order to find the right locations from the address. Even though their algorithms are quite efficient according to our manual checks, they cannot deal with all ambiguities and misspellings in the address data. For example, in several cases, the country code of, for example, Canada (CA) was interpreted as U.S. state code ‘California’ and the corresponding address was assigned to a location in the United States. This leads to wrongly assigned geocoordinates in some cases. The share of wrongly assigned geocoordinates is very difficult to estimate. After careful manual inspection of the output, we arrived at a list of common problems in the results from the APIs. Whenever such problems occur, it is very likely that the result is wrong or at least ambiguous:

1. The request yields another country than in the original queried address.
2. The postal code in the returned result is significantly different from the postal code in the queried address.
3. The request yields more than two results.
4. The request yields a location that is much more precise than the information in the queried address, (e.g., the queried address only contains a postal code and city, whereas the API gives a detailed address with street and house number).

Table 4 reports the prevalence of these problems. In addition to wrongly assigned data, the geolocalization yielded a total of 11.7 percent null results. These null results are distributed quite unequally across inventor and applicant countries, for example we obtained 2.7 percent null results for U.S. addresses, but 26.1 percent for Indian addresses. The geocoder seems to work very well with different languages (e.g., for Japanese addresses written in Japanese, we only obtained 2.5 percent null results). However, the geocoder yielded a high rate of null results for translated addresses (for example, addresses translated from Japanese to Latin characters that can be found in the PATSTAT data). The technical validation section discusses the validation of the geolocation. The Supplementary File 3 shows the proportion of non-null results for both inventor and applicant addresses we retrieved from PATSTAT in order to offer guidance for future data users (first column of the table). It should be noted, however, that the share of non-null results for Japan, China, and South Korea was much higher (above 97%) if we geocoded addresses in original languages (from the respective patent offices of those countries) rather than translated addresses from PATSTAT.

**Table 4:** Systematic geolocalization problems and their treatment.

Problem	Quantity	Treatment
The request yields another country than in the original queried address.	1.4% of all results	Correct country codes or use coordinates from other data sources
The postal code in the returned result is significantly different from the postal code in the queried address.	0.9% of all results from addresses with postal codes	Use coordinates from other data sources if API gave back a wrong location as likely consequence of wrong postal codes
The request yields more than two results.	1.3% of all results	Use coordinates from other data sources if the geolocalization service found more than two results
The request yields a location that is much more precise than the information in the queried address.	0.5% of the relevant results	Use coordinates from other data sources if the string length of the address in the geolocalization results was two to three times longer than the queried address.

#### Processing of geocoded results

We did process the geocoded addresses carefully in order to remove wrong, ambiguous, and null results. Given the large amount of data, we could of course only deal with systematic problems.

#### Extraction of postal codes

In order to process the data and to replenish information for wrong, ambiguous and null results, it was necessary to extract all available postal codes from the queried addresses using regular expressions in PostgreSQL. Because the postal code systems differ across countries, we had to implement the extraction country by country. For consistency, we had previously deleted all spaces appearing within postal codes in the cleaning procedures as described in Supplementary File 2. Here, we had to replenish all spaces and dashes at the correct positions in line with the respective postal code system. We also had to consider that if, for example, in a certain country a postal code has four digits, a four-digit number might also indicate something different such as a house number. We were able to identify most of the postal codes correctly by extracting them from the correct position from the address string where usually only postal codes appear in a specific country (e.g., in ‘1329 HAY STR WEST PERTH WESTERN 6005, AU’, the postal code is 6005 and not 1329). The extraction was more challenging for countries such as the United Kingdom where numeric and alphabetic digits appear in many variations (in the AA9A 9AA format).

As an example, we report the code for extracting postal codes for Brazil addresses in the format NNNNN- NNN:

SELECT ltrim(array_to_string(regexp_matches (address, '[0–9]{5}-[0–9]{3}', 'g'), '')) AS postal_code FROM addresses WHERE country = 'Brazil’;

In Portugal there was a change in postal codes in 1994 moving from four digits to 4 + 3 digits (NNNN-NNN). However, the first 4 digits are still the same so we extracted 4-digit codes if 4 + 3 digits were not available. In Brazil there was a similar change from 5-digits to 8-digits codes (NNNNN-NNN) in 1993 and we extracted 5 digits if necessary. In Germany, there was a change from 4-digit codes to 5-digit codes in 1993. The new codes have replaced the old ones completely and we could not link old to new codes. Therefore, we did not extract 4-digit codes at all. In sum, we could extract postal codes for 63.5 percent of all addresses. It should be noted that not all address fields have a postal code included, so the result from the extraction seems to be quite satisfactory.

#### Identifying problematic results

We continued with the data processing as follows: First, we checked results with countries that appeared in the geolocalization results, but should not have appeared there according to our initial selection of 46 countries. Some results corresponded to overseas territories. If they were close to the parent country, we kept the parent country. For example, we submitted

GODBYVAGEN 3 22100 MARIEHAMN, FI

into the API and received

3 Godbyvägen, Mariehamn 22100, Åland Islands.

In those cases, we have continued using Finland as inventor or applicant country. When the oversea territories were far away, we deleted the addresses completely from our database, e.g., for locations identified in Réunion or New Caledonia that are overseas territories of France in the Indian and Pacific Ocean, respectively. We also had some locations in our results, e.g., in the Bahamas or in Trinidad and Tobago, but those results were plainly wrong and referred to address fields that were very noisy and could not be correctly identified. If the queried addresses did not contain postal codes, we deleted them. With postal codes we might have had a chance of getting correct coordinates from other sources (see below).

In the next step, we scanned the results for the four common problems described above, see Table 4 for an overview. When we could identify one the problems we always tried to replenish information on coordinates from other data sources (mainly from the Geonames database, see section ‘Replenishment of problematic and null results’).

Regarding problem 1, we checked all cases where the country from the queried address differs from the country returned by the API. There were mainly two reasons for the discrepancy: First, the country in the queried address could have been wrong (very often due to inconsistencies in country and state codes used at the patent offices), but the API returned the correct country. Second, the geolocalization service assigned wrong locations, even in wrong countries. In these cases, we tried to get coordinates from other sources.

Regarding problem 2, we selected addresses where the first digit of the postal code extracted from the queried address is different from the first digit of the postal code given by the geolocalization service. Only if the differences are large enough (as in case of the first digit), we can be sure that the postal codes really belong to different locations so that the result must be wrong. However, for a few countries, different first digits still led to correct results (according to the street and city the API gave back as result in combination with coordinates). In those cases, we needed to add a further constraint when identifying problematic cases, namely that the city in the geolocalization results must not appear in the queried address.

Regarding problem 3, we selected the cases where the geolocalization service found more than two results. In case we obtained more than two results, we tried to get correct coordinates from other sources (primarily Geonames). In case we obtained only two results, we used the first one.

Finally, regarding problem 4, we compared the string length of the queried address with the address in the geolocalization results and we tried to get correct coordinates from other sources if they were significantly longer than the queried address. For 0.5 percent of the relevant results, the geolocalization yielded an address string that is more than twice as long as the queried address string. ‘Relevant’ means that only countries are considered where ‘twice as long’ is a good criterion and where the resulting addresses are not systematically longer. This could happen for example because the geolocalization service adds further information on regions to the address field or because it returns Latin letters where the queried addresses are written in Asian letters. We checked whether ‘twice as long’ is a good criterion by checking random samples for all countries. For a minority of countries, it was not a good criterion at all (so we did not apply it), for some other countries, the criterion had to be adjusted. For example, for Spain and Sweden we tried to get correct coordinates from other sources only if the address in the geolocalization results was more than three times longer than the queried address.

#### Replenishment of problematic and null results

We tried to replenish all problematic results that were identified according to problems 1 to 4 as well as the null results with coordinates from other data sources.

First, we replenished null results and problem 1 cases for USPTO patents with data from PatentsView (http://www.patentsview.org/download/). This service provides a list of cities with coordinates for USPTO filings. However, using this list we could only replenish location information for 1,373 locations that appear in 24,711 first filings (out of 16.2 million).

Second, we sought to match the extracted postal codes or city names for all problematic cases and null results with the list of postal codes and city names from Geonames and took coordinates from there. Geonames is a geographical database that contains over eleven million place names from all countries. It is available for download free of charge (https://www.geonames.org/export/). We applied a direct matching approach. According to He *et al*. (2018), approximate matching would increase the probability of generating false positives and would require considerable man and computer power^22^. Because the addresses for which we seek to retrieve coordinates may be ‘problematic’ in some sense (because they yielded null results or were associated with one of the problems described above), we did not want to introduce further ambiguities by applying approximate matching. However, we increased the likelihood that we could find a match considerably by matching on different combinations of columns found in Geonames.

We implemented the matching with data from Geonames in three stages. First, we looked for correspondence of the postal code and also searched for the city name from Geonames in the queried address. If the city could not be found in the queried address, the algorithm tries to match on another ‘admin name’ from Geonames. There are four admin names in the Geonames data reflecting different administrative levels such as regions, subregions, *etc*. About 37.3 percent of all address fields from the entire set of 7 million addresses could be matched on both the postal code and one of the city or administrative area names. Second, for cases where a search for a postal code together with a city/administrative area name was not successful (for example because of missing postal codes in the queried address or in case of old 4-digit postal codes in Germany), we matched on different combinations of administrative levels available in Geonames including the city. We started by matching on all admin level fields. If the matching is not successful, the algorithm looks for three out of four admin level fields in the address in different combinations. If the matching is still not successful, the algorithm looks for two fields and finally for only one of the fields. About 6.6 percent of all addresses from the entire set of addresses could be matched in this step. Third, for cases where the matching was still not successful, we only matched on the postal code. Even though the postal code appears to be a unique identifier of cities and districts, we decided to match on postal codes only in the last instance as some postal codes in the queried addresses have a wrong digit somewhere in the postal code. A further 16.6 percent of all addresses could be matched. For a small number of cases where both the postal code and city names are wrong, we could not assign coordinates. First matching on postal codes and searching for names is essential as some postal codes correspond to several cities or villages, especially in rural areas where several villages assemble into an association of municipalities. Overall, the success rate from matching on postal codes (with name search and without name search) and from matching on different combinations of administrative levels was 60.4 percent if applied on all available address fields.

Geonames does not provide information on postal codes for the following countries: China, South Korea, Greece, Estonia, Israel, Chile, and Bulgaria. To obtain coordinates for these postal codes, we took the average latitude and longitude for each postal code we obtained from the geolocalization results. Afterwards, we matched postal codes that we extracted from addresses that yielded null and problematic results (see above) on the postal codes available from geolocalization in order to assign average coordinates to the extracted postal codes and queried addresses.

We expect that the final dataset still contains some wrongly allocated addresses—even after our extensive pre- and post-processing. However, the potential effect of such mistakes on research results will be negligible given the large number of addresses. Furthermore, some mistakes may be irrelevant depending on the research purposes. For instance, when it comes to the measurement of inventiveness of geographic regions, it does not matter that we might have locations that are too (falsely) precise as long as they are in an area that lies in the same region as the correct location. After manual examination of thousands of results, we have concluded that the problem of mis-assignments is negligible compared to the number of correctly assigned locations.

### Stage 3: regionalization

The assignment of regions and cities to the information on longitude and latitude is a central element in the data generation process. Researchers and policymakers are often interested in outcomes at aggregated, but still fine-grained levels, e.g., the number of patent applications filed by inventors in a certain region or city. The information on administrative areas comes from GADM data (https://gadm.org/data.html, version 3.6 from May 2018). The website provides spatial data on 386,735 administrative areas worldwide as a shapefile. We imported the file into a geospatial database (PostGIS) and assigned latitudes and longitudes from our database to the correct cities and regions (after all data processing had been completed). GADM provides the country name in variable name_0, and administrative regions and cities in name_1 to name_5. The countries vary with respect to the number of available administrative regions. Usually, the city level can be found in either name_3 or name_4. For all countries we checked at which level the city is available. An extra column city using this information has been added. In some countries, the largest city appears to have a structure different from other cities because the most fine-grained administrative area displays city districts. We took this into account and report in the city level column the city name (e.g., Paris) rather than districts (e.g., Paris, 11e arrondissement). For Chinese cities, the assignment was more complicated because of the large variety of terms for lower-level administrative units (‘County City,’ ‘Municipality,’ ‘Prefecture City,’ etc.).

We checked the information from GADM against the information on administrative areas that is given in the geolocalization results and in the Geonames data. However, as the administrative regions sometimes appear unsystematically in these data, we decided to only report data from GADM. In few cases (for about 900 locations), the latitudes and longitudes could not be assigned to a region and city—very often corresponding to locations on the sea-earth border. For 100 locations with the highest number of filings, we assigned the correct administrative regions manually (e.g., for Zhuhai Shi in China or Shinagawa in Japan). In the remaining 800 cases, the number of filings is relatively small so we do not think it will affect any research results.

Given the information from GADM, we are able to count the number of first filings not only by country, but also by region and city—this possibility had been only available for a very selected set of patents and regions so far.

### Stage 4: final data assembly

The dataset contains geographic data on first filings. These are the first filings within a family of patents and are thus closest to the invention date. Building a database of first filings with location information on inventors or applicants is challenging because many applicant and inventor addresses are missing for the respective patent document. This is due to the fact that first filings are registered at various patent offices and that the rules of registering addresses vary.

We applied the algorithm from de Rassenfosse *et al*. (2013) in order to impute missing information from within the patent family. We first updated their algorithm that was developed for country codes in PATSTAT and run it with newer PATSTAT versions (PATSTAT autumn 2016 and PATSTAT spring 2019). In a next step, we applied it on our newly developed database with coordinates from the geolocalization, PatentsView and Geonames. We adapted the algorithm so that it can impute more detailed location names and coordinates if the information is missing for the respective first filing. For this, we had to assign application identifiers from PATSTAT (appln_id from table TLS201_APPLN) to all longitude/latitude pairs that identify unique locations.

We extended the pool of ‘pure’ priority filings by including all possible patent applications that have been filed at one of the patent offices for the first time and refer to them as first filings. First, we use all priority filings as defined in the strict sense: These are so-called Paris Convention priorities of an application that can be selected via the identifier prior_appln_id from PATSTAT table TLS204_APPLN_PRIOR. The Paris Convention for the Protection of Industrial Property allows the applicant of a first application filed in one of the contracting states to seek protection in any of the other contracting states within a period of 12 months. However, using only this table of priorities, we would miss patent applications filed under the PCT that are not Paris Convention priorities. The PCT makes it possible to seek patent protection in a large number of countries simultaneously. For this purpose, the applicant has to file a single international application either at the WIPO or at a national or regional patent office such as the EPO. Consequently, we added PCT filings to our pool of first filings (international applications and the receiving patent offices can be identified in PATSTAT table TLS201_APPLN). Finally, we can identify two other kinds of first filings: ‘Parent applications’ of so-called ‘Application continuations’ and filings based on ‘Technical relations’ that define some kind of family-relationship between patent applications that have not been published by the patent office. They can be identified from PATSTAT tables TLS216_APPLN_CONTN and TLS205_TECH_REL. The PATSTAT data catalog offers technical definitions and more details^23^.

In a similar vein, we built a pool of all subsequent filings that refer to the first filings. Usually, these subsequent filings are applied for in other jurisdictions than the first filing. In case of PCT applications, we refer to information from the National or Regional Phase where the applicant seeks protection at national or regional offices that are different from the office where the international application has been filed first. We can identify subsequent filings for all kind of first filings from the tables mentioned above (e.g., in TLS204_APPLN_PRIOR, appln_id identifies subsequent filings).

The general rule for the imputation of missing information is:

Always prefer information from the first filing: If the information is not available look into the family for subsequent filings and use information from the first equivalent (otherwise look for the information in other subsequent filings within the family).

To be more precise, the algorithm first selects all available location information (including information on latitude and longitude, city, and administrative divisions) that is available from the first filing itself. For each filing that has missing information on the inventor’s (applicant’s) location, the algorithm looks into five additional sources of information. The sources are browsed subsequently in order to retrieve missing information. The algorithm stops looking into those sources, once it has found the information in one of them. Source 1 is the first document itself, whereas sources 2 and 3 exploit family linkages. Sources 4 to 6 look into location information of applicants, thereby assuming that the applicant’s address is likely to be close enough from the inventor’s address. The following list provides more detailed information on the sources. If the interest lies in the applicant’s location rather than the inventor’s location, the sources are browsed the other way around starting with the applicant’s address in the first document (as indicated in parentheses for each source).

- Source 1: Uses the inventor’s (applicant’s) location from the first document itself.
- Source 2: If no information on the inventor’s (applicant’s) location is available from the first document, the earliest direct equivalent is browsed. A direct equivalent is a second filing claiming the first application in source 1 as sole priority.
- Source 3: If no information on the inventor’s (applicant’s) location is available in the direct equivalents, the other second filings of the same family are browsed. The second filings considered in this source claim more than one first document.
- Source 4: If no information on the inventor’s (applicant’s) location is available in the other subsequent filings, the applicant’s (inventor’s) location from the first document is used.
- Source 5: If no information on the applicant’s (inventor’s) location is available from the first document, the earliest direct equivalent is browsed for this information.
- Source 6: If no information on the applicant’s (inventor’s) location is available in the direct equivalents, the other second filings of the same family are browsed.

Regarding PCT filings, it is not possible to distinguish between direct equivalents and other subsequent filings. We therefore use information from the Regional Phase as Source 2 and information from the National Phase as Source 3. We prefer information from the Regional Phase because regional offices such as the EPO provide relatively complete address data compared with some national offices. The technical validation section discusses the validation of the imputation.

## Data Records

### Data and variables

The resulting datasets geoc_inv.txt and geoc_app.txt are available from the Harvard Dataverse repository (10.7910/DVN/OTTBDX)^24^. The file geoc_inv.txt contains identifiers for first filings (corresponding to appln_id in PATSTAT), latitude, longitude, city, region, and country of the inventor. The file geoc_app.txt is similarly structured. It contains application identifiers for first filings (appln_id), latitude, longitude, city, region, and country of the applicant.

Missing coordinates have been imputed from equivalents and other second filings or from information on the location of applicants (see above “Final data assembly”). Due to potential privacy issues, we provide latitudes/longitudes in truncated format in the dataset that is available in the repository. More specifically, they are stored in PostgreSQL’s inexact data format REAL, meaning that they contain three to four decimal places. However, the level of precision should be sufficient for most localization tasks.

Both files also contain a variable indicating the source of information (source), and the source of coordinates (whether they come from geolocalisation services, from Geonames, or from PatentsView). It is possible to select certain types of first filings based on column type. For example, Paris Convention priority filings can be retrieved by specifying type = priority.

The variables are listed in Table 5.

**Table 5:** List of variables.

Variable name	Description
appln_id	application identifier from PATSTAT, which identifies the first filing
patent_office	patent office where the first filing was filed
filing_date	filing date of first filing
lat	latitude
lng	longitude
ctry_code	country code
name_0	country
name_1	1^st^ administrative area
name_2	2^nd^ administrative area
name_3	3^rd^ administrative area
name_4	4^th^ administrative area
name_5	5^th^ administrative area
city	city name (in the United States: county name)
coord_source	geolocalization: information on latitude and longitude comes from a geolocalization web service geonames: information on latitude and longitude comes from geonames.org patentsView: information on latitude and longitude comes from PatentsView
source	source where information on latitude/longitude comes from
	1: information comes from the first filing itself
	2: information comes from direct equivalent
	3: information comes from other subsequent filings
	4: information comes from the applicant’s location in first filings
	5: information comes from the applicant’s location in the equivalent
type	6: information comes from the applicant’s location in other subsequent filings
	priority: first filing is a Paris Convention priority pct: first filing is an international application (not claiming a Paris Convention priority filing) continual: first filing is a parent filing (and not a Paris Convention priority) tech_rel: first filing is based on a technical relationship (and not a Paris Convention priority)
	single: singletons, i.e. filings without further family members (and thus without subsequent filings)

The dataset geoc_inv.txt includes 18.8 million geolocalized first filings with filing year between 1980 and 2014 out of a total number of 23 million first filings (information from the JPO and CNIPA is only available until 2013). The corresponding number for geoc_app.txt is very close.

#### Counting first filings by inventor countries

**Fig. 2:**
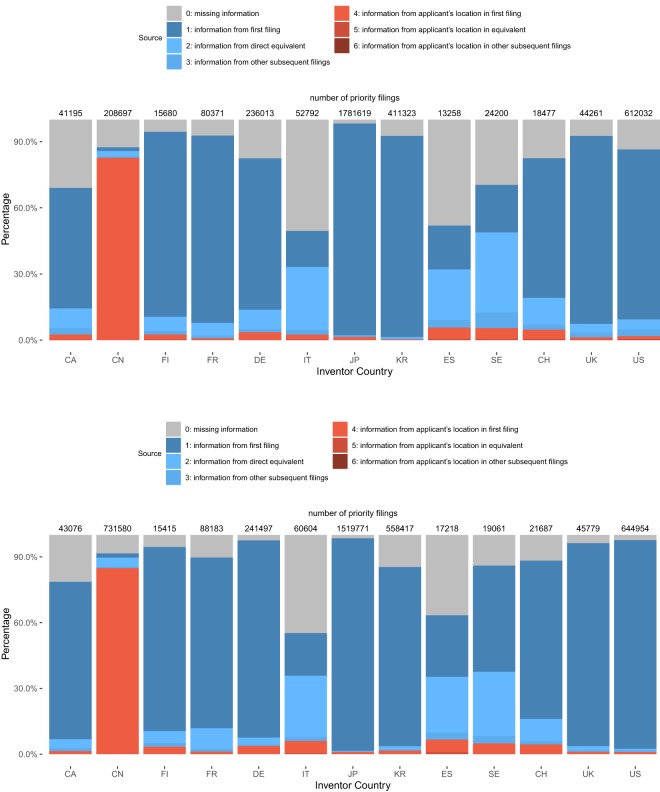
Share of information retrieved from different sources for selected inventor countries (upper panel: filing years 2000 to 2004; lower panel: filing years 2005 to 2009).

Figure 2 shows the breakdown of sources used to obtain information on latitude/longitude for each first filing for a selection of inventor countries with the highest number of first filings. It reports two time periods: application years 2000–2004 and 2005–2009. As described above, source 1 means that we have used information contained in the original patent document and 2–6 means that the information on location has been imputed (please see Section ‘Stage 4: Final Data Assembly’ for the detailed description of the sources). In this figure, a value of ‘0’ means that the information is still missing and could not be imputed. The estimated number of first filings is displayed on top of each bar. The figures allow an assessment of the coverage of the data. The number of filings from source ‘0’ is not directly available in our dataset and comes from prior work performed by de Rassenfosse *et al*. (2013) at country code level that has been updated with a more recent PATSTAT version. In this prior work, country codes that were still missing after browsing sources 1 to 6 were set equal to the patent office’s country. In addition, the coverage only using data at country code level is better—source 0 thus refers to country codes that can be found following the approach in de Rassenfosse *et al*. (2013), but the respective first filings are not in the data at hand due to missing exact location data.

For 2005 to 2009, the location information for inventors in China, Japan and the United States is very complete thanks to the data we have directly from the USPTO (via PATSTAT), JPO and CNIPA (given that most of the first filings by U.S., Chinese and Japanese inventors are filed at the respective country’s office). However, for China we could only geocode a fraction of inventor addresses, i.e. those that were already in PATSTAT. All other information we have from CNIPA relates to applicant addresses, which is the reason why almost all information comes from source 4 for inventors. For Germany, France and the UK we also have a good coverage thanks to the data from the national patent offices. In those countries source 2 is used to a higher degree. For German inventions, we know that many patent applications are filed at the EPO for which we also have location data included, so most information either comes from first filings at the EPO or from second filings at the national office (or conversely, from first filings at the national office and second filings at the EPO). For smaller countries such as Switzerland, a relatively large share of information can be retrieved from second filings even though we did not include information from those countries’ patent offices. However, smaller countries are more likely to file patent applications at larger foreign patent offices first so that the location can be retrieved from the DPMA or EPO, for example. The coverage for Canadian and South-European inventors is less satisfactory, but seems to be still acceptable. Of course, the inclusion of information from those countries’ patent offices would have improved the overall picture.

Overall, the coverage has improved over time (compare Panel 1 and 2 in Fig. 2). Columns 2 and 3 in the table in the Supplementary File 3 provide information on the coverage for all inventor countries (aggregated over all sources). Column 4 shows the share of first filings by inventor country in the total number of first filings (again based on de Rassenfosse *et al*. (2013) country data) showing that the bulk of filings goes back to Japan, China, the U.S., South Korea, and Germany for which the coverage with exact location data is particularly good. The coverage is worse for more “peripheral” countries that are probably less integrated in the international patent system (however, they only account for a tiny share in total patenting activities).

## Technical Validation

There are three critical aspects to our workflow that could compromise the validity of the results: mistakes in the pre-processing of addresses; mistakes during the geocoding of addresses and post-processing; and mistakes during the imputation of missing data on first filings. We discuss these issues in turn.

### Validation of pre-processing and geocoding

In order to test the quality of the final result with respect to pre-processing and geocoding, we fed a random sample of 1,000 unprocessed raw addresses from PATSTAT that is stratified according to the number of addresses by inventor country to a geolocalization service based on OpenStreetMap. When the distance between the coordinates from OpenStreetMap and the coordinates from our data was smaller than 5 km, we considered them as being sufficiently close. Excluding all cases where one of the geolocalization services gave a null result (corresponding to 318 cases, suggesting that the geolocalization services do not cope well with unprocessed addresses), left us with 635 addresses. Comparing the test output with the results from geocoding of fully processed addresses, we found 114 cases where the discrepancy between the coordinates was in excess of 5 km. We checked those cases manually in order to find reasons for the discrepancy. It turned out that 75 locations in the processed results where correct compared with 41 in the unprocessed results. Two locations were correctly identified in both the test and processed output, but the distance was slightly larger than 5 km because the location is a rather broad U.S. county and thus not very exact. Two results in our data could be replenished with information from Geonames leading to 77 correct processed results. The other results were ambiguous (28 in the test output, 25 in the processed results) or wrong (45 in the test output, 12 in the processed results). The main source of ambiguity was city names without any further information showing up in the address field. For example, for PLAINFIELD, US, one geolocalization service gave back Plainfield, Will County, Illinois, USA as result, whereas the other gave Plainfield, Indiana, USA. Without further information, it is impossible to decide which result is correct. The problem of ambiguous city names also affects the processed results. If the numbers from the manual check can be generalized to the whole database, 2 percent of the results could be probably wrong and up to 4 percent could be considered ambiguous.

We did some further checks with respect to a selected set of patents, namely patent applications filed at the EPO and at the USPTO, respectively. We draw a random sample of each 100 inventor addresses from EPO patents in REGPAT and from USPTO patents in PatentsView (stratified according to the number of addresses per inventor country) and compared the region (for USPTO: city and region) with the location in our address dataset (after processing of results). In sum, we found 98 out of 100 REGPAT patents in our data. We checked the cities and regions in REGPAT and in our data manually based on the raw address field. We assigned cities and regions correctly for 85 EPO patents. About 5 cities were not completely accurate, but very close to the correct location. For example, we assigned the administrative areas Île-de-France (Region), Val-de-Marne (Department), L'Haÿ-les-Roses (Arrondissement), Cachan (Commune) to one of the EPO patents, but the city in the raw address is Arcueil, not Cachan. However, Cachan is in the direct neighborhood of Arcueil—both are towns in the south of Paris. The reason for such mis-assignments is perhaps accuracy problems in the coordinates or in the shape file, but such little discrepancies are of course negligible. Finally, 8 cities and regions were not correct, i.e. the geocoding of the address must have failed. A direct comparison with REGPAT shows the following: First, REGPAT seems to be of similar high quality with only few mis-assignments of regions. Second, the main problem in REGPAT is that they could not assign regions in all cases (because they do not have coordinates). In contrast, we were able to assign administrative areas—and even cities—whenever we had coordinates. Third, a further problem is that REGPAT often displays very broad regions such as Tokyo or New York-Newark-Bridgeport to a patent that are metropolitan areas with extremely large numbers of both inhabitants and inventors. In contrast, we are able to display more fine-grained areas such as cities or counties. For large metropolitan areas such as Tokyo or Paris, we are even able to go down to district level (e.g., Tokyo, Ōta or Paris, 7e arrondissement), and of course, it is possible to use the coordinates that correspond to the most precise level of localization. In sum, for 12 addresses, regions in the REGPAT sample were not correct or not available at all, but as another method and definition of regions were used for the REGPAT data generation those wrong cases are not directly comparable with those we found in our data.

The PatentsView data is similar to ours as they also contain coordinates, cities and regions or states. The only difference is that they only contain data from one patent office (USPTO). It turned out that the PatentsView data have many mistakes which probably goes back to errors that happened in the geocoding. We found that 47 locations in our random sample from PatentsView were wrong, 14 even showed a location in a wrong country. In contrast, if we look at the same USPTO patents in our data, we find the proportion of correctly geocoded and regionalized patents similar to the ones being reported above for EPO patents and for PATSTAT addresses.

### Validation of imputation

Regarding the imputation of missing data, the algorithm has been validated extensively by de Rassenfosse *et al*. (2013). However, as we streamlined the algorithm, adapted it to PostgreSQL and run it with many more parameters, we also checked a random sample, stratified according to the number of first filings with imputed location information by inventor country and used sources (sources 2 to 6). We checked the assigned city with information we found in original patent documents in Espacenet (https://worldwide.espacenet.com), which is a patent search engine hosted by the EPO with access to over 100 million patent documents. We translated the inventor or applicant addresses with Google Translate if necessary.

Consider the following case to illustrate: use of source 2 for an INPI first filing with an equivalent DPMA patent. In that case, we first checked the location information in our data for correctness by looking into the INPI document and then the DPMA patent in Espacenet. In many cases, the address was indeed not available in the first filing document, but in the second filing document. In some other cases, the address was actually available in the first filing document, but is was not in our address database (due to gaps in the raw data that is made available in PATSTAT or in the other data sources we have used). In those cases, we checked the correctness of the imputation from other sources by directly looking at the address in the first filing original document and comparing it with the location retrieved from other patent documents. We checked about 90 patent documents using information from sources 2 to 6 in this way. We did not come across mistakes regarding the imputation of information from subsequent filings, i.e. the address information was correctly retrieved in all cases. Thus, remaining mistakes (i.e., wrong location information) solely result from wrong geocoding (see discussion above), but not from the imputation from information in the patent family.

## Usage Notes

We recommend importing our data into a relational database where the data can be joined with PATSTAT tables. For all users that do not have access to PATSTAT or want to work with software such as Stata or R, we provide an additional bridge file first_and_subsequent_filings.txt. It is available from the Harvard Dataverse repository https://dataverse.harvard.edu/dataset.xhtml?persistentId=doi:10.7910/DVN/QLT9WM ^25^.

The purpose of the bridge file is to assign patent publication numbers of all filings within a family to the respective first filing. The information is derived from PATSTAT tables TLS201_APPLN, TLS204_APPLN_PRIOR, TLS216_APPLN_CONTN, TLS205_TECH_REL, and TLS211_PAT_PUBLN. Note that the files geoc_inv.txt and geoc_app.txt only contain first filings. First filings can be identified via the variable prior_appln_id in TLS204_APPLN_PRIOR, tech_rel_appln_id in TLS205_TECH_REL, parent_appln_id in TLS216_APPLN_CONTN, and appln_id in TLS201_APPLN (for international applications, internat_appln_id = 0 must hold, see PATSTAT data catalog). In our data, all first filings have the uniform variable name appln_id.

To illustrate, consider patent number US2009212767A1 that has appln_id = 266850837 in PATSTAT. This patent document is not in the geolocalized data because it is not a first filing. Instead, it is a subsequent filing of EP1850096A1 having appln_id = 104 in our data. In the file first_and_subsequent_filings.txt, we created a dummy variable is_first that takes on value 1 whenever the respective patent number corresponds to a first_filing (0 if it is a subsequent filing). Looking into the file first_and_subsequent_filings.txt, one can see that for EP1850096A1, is_first is 1, meaning that this entry corresponds to the first filing within a family. In contrast, for US2009212767A1, the field is_first is set to 0, but appln_id is still 104. This indicates that US2009212767A1 is a subsequent filing of 104.

If a researcher wants to merge her dataset with patent publication numbers (and this dataset might contain subsequent filings as well), she should proceed as follows:

1. Merge the data with first_and_subsequent_filings.txt on the patent number that usually consists of publn_auth, publn_nr and publn_kind. If available, use publn_nr_original rather than publn_nr. Note that for data from the USPTO (or PatentsView), it is usually sufficient to use publn_nr_original or publn_nr, i.e., for US2009212767A1 use 2009212767. In PatentsView, publn_nr is called patent_id. For other jurisdictions, it is most often necessary to merge on the concatenated columns (concat(publn_auth,publn_nr,publn_kind)).
2. Merge the data with geoc_inv.txt or geoc_app.txt on the variable appln_id.
3. Now the location information from the first filing should be available for all patent numbers in the data including all subsequent filings (provided that there is location information for the respective first filing in the present data).
4. For further details, refer to the PATSTAT Data Catalog (https://www.epo.org/searching-for-patents/business/patstat.html#tab-3).

When using the data, please pay attention to inventor locations that are associated with a high number of patents. This is potentially an indication that the inventor address reported in the patent document does not relate to her place of residence but rather to her place of work. One way to find out is to check whether the applicant and inventor locations overlap. If they do, the inventor address probably indicates the place of work.

## Supplementary information

Supplementary File 1

Supplementary File 2

Supplementary File 3

## Data Availability

The data extraction and parsing were done in Python 3.4 and 3.6. The construction of address fields, the cleaning, data processing, matching with Geonames, and imputation of missing information was implemented in PostgreSQL 9.6.6. The assignment of regions and cities was done in PostGIS 2.4. All Python and PostgreSQL code produced for this project can be accessed upon request. The PostgreSQL codes for the imputation of missing country codes and location information is available on Github (https://github.com/seligerf/Imputation-of-missing-location-information-for-worldwide-patent-data).
