# Supplementary material for: Geocoding of worldwide patent data
Source: Sci Data. 2019 Nov 6;6:260. doi: 10.1038/s41597-019-0264-6 (PMC6834584; doi:10.1038/s41597-019-0264-6)
Supplement: Supplementary file 1 [file 41597_2019_264_MOESM1_ESM.pdf]

Supplementary information

Set of heuristic rules applied in the cleaning procedure

- Change all characters to upper cases and delete all non-alpha numeric characters
- Delete all characters that do not belong to an address such as trailing zeroes or single characters
- Delete all addresses that only contain a country code
- Standardize `'ß'` to `'ss'` and the following characters:
  `'áàâãäåāăąÀÁÂÃÄÅĀĂĄçèééêëēĕėęěĒĔĖĘĚÉÊÈìíîïìĩīĭÌÍÎÏÌĨĪĬóôõöōŏőÒÓÔÕÖŌŎŐØùúûüũūŭůÙÚÛÜŨŪŬŮýřšŠČÇĆÑŽÆæŸñøŃŻğışİŞžŁÝý'`
  to
  `'aaaaaaaaaaaaaaaaaaceeeeeeeeeeeeeeeeeeiiiiiiiiiiiiiiiioooooooooooooooouuuuuuuuuuuuuuuuyrssccc nzaaynonzgisiszlyy'`.
- Delete trailing spaces or consecutive spaces occurring in the string or at the end
- Delete terms related to numbers and postal codes:
  (POSTAL|POSTFACH|ZIP|PIN|POST|CODE|DIVISION|N°|NO|NR|NUMBER)
- Delete terms and abbreviations related to rooms, buildings and apartments and the consecutive numbers:
  (MAH|MAHALLESI|DAIRE|APARTMANI|APARTMAN|CASA|ESC|PTA|PUERTA|HOUSE|PLOT|FLOOR|AP|APP|APT|APARTM|APARTMENT|APARTMENTS|APPARTEMENT|APARTAMENTO|APARTADO|APTD|WOHNUNG|WHG|POSTBUS|APTO|APPT|APT|FLAT|ROOM|ETAJ|EPULE|CAMPUS|SUITE|ENT|ENTR|ENTRANCE|ET|BLOC|BLOCK|BLOK|BLK|SECTION|SECT|FL|BUILD|BUILDING|BLDG|BID|BL|BLD|BD|BATIMENT|BAT|BI|DOOR|VH|DEPARTMENT|DEPT|DEP|DEPARTAMENTO|DEG|KOMPLEX|COMPLEX|KOMPL|RESIDENCE|EXT|EXTENSION|BOX|BOITE|BTE|TRAKT|SECTORULED|EDIF|EDIFICIO|)
- Delete terms used for regions (often used inconsistently):
  (RAJ|RAJONS|COUNTY|REGION|REG|DIST|DISTRICT|DISTRITO|FEDERAL|CENTRAL|REG|REPUBLIC OF|REPUBLIC|FEDERATION|PROVINCE OF|PROVINCE|PROV OF|PROV|STATE|ZONE|AREA|KANTON|CANTON OF|CANTON DE| CANTON|PREFECTURE|ESTADO DE|ESTADO|EDO)
- Delete terms related to the legal status of companies or the type of activity:
  (GMBH|AG|INDUSTRIAL|INTELLECTUAL PROPERTY| IP|CO|LIMITED|LTD|LTDA|HOLDING|INDUSTRIELLE| INDUSTRIAL|PATENT|GROUP|LABORATORIES|INC|CORPORATION|CORP)
  In addition to that, we also used a more comprehensive list from NBER Patent Data Project in order to delete terms related to company names in many languages (https://sites.google.com/site/patentdataproject/Home/posts/namestandardizationroutinesuploaded).
- Delete country names that appear in different languages within the same country (e.g. 'SWITZERLAND', 'SCHWEIZ', 'SUISSE')
- Delete country codes if they appear within an address and not at the end
- Delete abbreviations and stop words that appear systematically in addresses in specific countries, e.g.,
  (AN DER|IN|STOCK|DCHA|POL|IND|INDS|CV|COL|DRT|CERTO| ESQUERDA|ESQ|°|DTO|ZAM|DF|PEOPLE|CEP|CPF|AP|ESTACION|SOK|SIT|KAT|MAH|MH|KM|PK|CG|CGH|BEI|AM|SL|SLO|ZG|KWARTIRAPOS|MO|CT|TH|RTM|UT|AD|OT|NRW|NTN|SNR|NXP|TER|JL|HML|HKV|VR|KI|VTT|ESTONIA|BL|JUDET|JUD|JUDETUL|RM|ET|SC|LOTE|ISRALCO|FT|FIN|KV|KW|ZH|SF|CP|CR|CS|DV|RR)
- For consistency, use abbreviations in the following cases and make sure that there is a whitespace between the abbreviation and the preceding term:
  STREET -> STR,

```
STRASSE -> STR,
BOULEVARD -> BLVD,
BOULD -> BLVD,
AVENIDA -> AV,
AVENUE -> AV,
ROAD -> RD,
ALEJA -> AL,
OSIEDLE -> OS
CERRADA -> CDS,
SAINT -> ST,
ULITSA -> UL,
PROSPECT -> PR,
PROSPEKT -> PR,
CADDESI -> CD.
```

The cleaning also involved several country-specific cleaning procedures (with respect to the country of inventor or applicant, not the country of the patent office). First, we noticed an inconsistent use of abbreviations vs. full names for administrative areas such as counties, regions and states. We have harmonized the addresses by using abbreviations to designate states instead of full names for Australia, Canada and the United States. For example, occurrences of 'VICTORIA' in Australian addresses were systematically changed to 'VIC'. Second, there is also an optional use of regions in addresses of some countries. We systematically dropped region identifiers for Austria, Chile, India, Ireland, New Zealand, Poland, South Africa, Russia, Italy, Mexico, Spain, Switzerland, Turkey, and Great Britain. For example, in Austria, the state ‘TIROL’ was deleted in all addresses where it showed up.

Next, we ensured that large cities do not appear in different languages and variants of spelling. For example, for Brussels, we only used the variant in French, i.e. we set 'BRUSSEL', 'BRUESSEL', and 'BRUSSELS' to 'BRUXELLES'. In the case of Mexico City, we found many different variants that all refer to the District Federal which is equal to Mexico City. We have made sure that only ‘MEXICO CITY’ is used and changed the names as follows:

```
'MEXICO D F', 'MEXICO DF', 'MEXICO DISTRITO FEDERAL', 'MEXICO DISTR FED', 'MEXICO DISTRICT FEDERAL', 'MEXICO CITY', 'D F', 'DISTRITO FEDERAL', 'DISTR FED', 'DISTRICT FEDERAL' -> 'MEXICO CITY'.
```

Finally, whenever a country has postal codes that have a space between the postal code’s digits according to official guidelines (e.g., in Sweden the official format is NNN NN), we deleted the spaces because we could always find numerous addresses where the postal codes did not have a space where they should (in Sweden, many postal codes appeared as NNNNN). Thus, we have made sure that postal codes appear in the same way in all addresses even though the format is not correct (but this does not affect the outcome of geocoding).

We applied a number of additional procedures to data from specific patent offices. Regarding DPMA, we deleted academic titles such as “DR”, “ING”, “DIPL”, “PROF” that are commonly used in Germany and showed up in the inventor fields. The position of abbreviations for region names within the DPMA address string was quite inconsistent (as described above, abbreviations are commonly used in Australian, Canadian, and U.S. addresses) so we moved all of them to the end of the address string whenever we were able to detect them automatically. For DPMA’s U.S. addresses, we came up with an involved regular expression that can distinguish between cities and states (this matters for entities such as “NEW YORK” or “WASHINGTON”, which are both states and cities) in order to get the correct address field with regions at the desired position. For the data coming from the Asian patent offices, due to the complexity of the Chinese (Hanzi), Korean (Hangul) and Japanese (Kanji) symbols, we could only apply some basic cleaning procedures such as deleting unnecessary spaces or “noisy” and special characters. In the JPO inventor address table, we deleted the second substring of the address field (as

indicated by a space between the first and the second substring) because the second substring always contains a company or institution name rather than the address information. For example, in the following address

大阪府大阪市阿倍野区長池町２２番２２号　シャープ株式会社内

the last substring can be translated into “Sharp Corporation”.

In the CNIPA data, we identified terms such as

经济开发区, economic development zone

工业区, industrial area

高新区, high-tech zone

高新技术开发区, high-tech development zone

工业园, industrial area

经济区, economic zone,

and deleted them because they do not help geolocation.
