# Supplementary material for: Geocoding of worldwide patent data
Source: Sci Data. 2019 Nov 6;6:260. doi: 10.1038/s41597-019-0264-6 (PMC6834584; doi:10.1038/s41597-019-0264-6)
Supplement: Supplementary file 2 [file 41597_2019_264_MOESM2_ESM.pdf]

Table S1. Systematic comparison of the present data with the literature, along a selected number of dimensions

	**Time Period**	**Number of patents**	**Basic entity**	**Reference unit**	**Granularity**	**Patent offices**	**Purpose**	**Method**
The present work	1980-2014	18.8 mio.	PATSTAT application id (first filing)	Applicant and inventor coordinates	Actual place or city level	52 patent offices	Localization of inventive activities	Geocoding
OECD REGPAT (July 2019)	1977-2019	6.7 mio.	Patent number and PATSTAT application id	Applicant and inventor regions	NUTS3 or TLS3 regions	EPO + WIPO	Regionalization of patent data	Regions allocated based on postal code or town names
de Rassenfosse et al. (2013)	1980-2013	18.1 mio.	PATSTAT application id (first filing)	Inventor countries	Country level	52 patent offices	Measuring inventive activities at country level	Identification of country code
PatentsView	Since 19th century (not complete)	6.9 mio.	Patent number	Applicant and inventor coordinates	City level	USPTO	Localization of inventive activities	Unknown
Morrison et al. (2017)	1975-2011	8.5 mio.	Patent number	Applicant and inventor coordinates	Place or city level	USPTO + EPO + WIPO	Name disambiguation	Geocoding
Li et al. (2014)	1975-2010	4.2 mio.	Patent number	Inventor coordinates	City level	USPTO	Name disambiguation	Coordinates allocated from official sources
Regional data in PATSTAT	1978-2018	1.7 mio.	PATSTAT application id	Applicant and inventor regions	NUTS3 regions	EPO	Regionalization of patent data	Regions allocated based on postal code or town names
