# Supplementary material for: Geocoding of worldwide patent data
Source: Sci Data. 2019 Nov 6;6:260. doi: 10.1038/s41597-019-0264-6 (PMC6834584; doi:10.1038/s41597-019-0264-6)
Supplement: Supplementary file 3 [file 41597_2019_264_MOESM3_ESM.pdf]

Table S3. Share of non-null results from geolocalisation of PATSTAT addresses (inventor and applicant countries) **(1)**; share of first filings by inventor country for which detailed location information is available or has been imputed, 1990-1999 **(2)**; share of first filings by inventor country for which detailed location information is available or has been imputed, 2000-2009 **(3)**; share of first filings by inventor country in the total number of first filings **(4)**

**Country**	**(1)**	**(2)**	**(3)**	**(4)**
United States	97%	88%	92%	12.45%
Netherlands	96%	67%	81%	0.27%
Poland	96%	12%	17%	0.44%
Australia	96%	16%	34%	0.43%
Germany	95%	82%	90%	5.42%
Austria	95%	68%	83%	0.19%
Slovenia	95%	20%	47%	0.02%
Lithuania	94%	4%	27%	0.01%
Belgium	94%	81%	87%	0.14%
Slovakia	93%	11%	29%	0.02%
Czech Republic	93%	13%	36%	0.07%
Italy	93%	39%	53%	0.84%
Estonia	93%	57%	83%	0.01%
Finland	92%	92%	95%	0.28%
Switzerland	92%	79%	86%	0.36%
Hungary	92%	27%	40%	0.25%
Canada	91%	62%	74%	0.72%
New Zealand	91%	21%	45%	0.08%
Croatia	90%	12%	30%	0.01%
Mexico	90%	18%	42%	0.04%
Luxembourg	90%	69%	80%	0.01%
France	90%	86%	91%	1.45%
Latvia	90%	8%	28%	0.02%
Denmark	90%	75%	83%	0.12%
Liechtenstein	89%	58%	59%	0.01%
Romania	87%	3%	15%	0.22%
Sweden	87%	72%	77%	0.41%
Brazil	87%	6%	17%	0.26%
Norway	85%	53%	64%	0.10%
Chile	83%	25%	54%	0.01%
Spain	83%	35%	58%	0.25%
Portugal	82%	18%	57%	0.02%
India	82%	34%	82%	0.30%
Israel	82%	74%	89%	0.20%
Ireland	81%	69%	83%	0.05%
United Kingdom	80%	92%	95%	0.76%
Turkey	79%	5%	31%	0.04%
Greece	79%	12%	27%	0.04%
South Africa	77%	11%	18%	0.14%
Russia	76%	2%	4%	2.66%
Iceland	75%	52%	73%	0.003%
Bulgaria	69%	10%	27%	0.12%
China	65%	51%	91%	21.81%
Japan	60%	98%	98%	40.42%
Malta	58%	40%	77%	0.001%
South Korea	33%	63%	88%	7.55%
Others				0.98%
